# Validity, responsiveness and minimal important change of the EQ-5D-5L in patients after rotator cuff repair, shoulder arthroplasty or thumb carpometacarpal arthroplasty

**DOI:** 10.1007/s11136-021-02849-7

**Published:** 2021-05-10

**Authors:** Miriam Marks, Cécile Grobet, Laurent Audigé

**Affiliations:** Department of Teaching, Research and Development, Schulthess Klinik, Lengghalde 2, 8008 Zurich, Switzerland

**Keywords:** Measurement properties, Psychometric properties, Quality of life, Upper extremity, Hand

## Abstract

**Purpose:**

The aim was to investigate the measurement properties of the EQ-5D-5L utility index in patients after arthroscopic rotator cuff repair (RCR), total shoulder arthroplasty (TSA) or thumb carpometacarpal (CMC I) arthroplasty.

**Methods:**

In this prospective study, all patients completed the EQ-5D-5L before surgery and 6 months and 1 year after surgery. In addition, RCR patients completed the Oxford Shoulder Score (OSS), TSA patients completed the Shoulder Pain and Disability Index (SPADI) and CMC I patients completed the brief Michigan Hand Outcomes Questionnaire (brief MHQ) at each designated time point. Construct validity (Pearson’s correlation coefficient, r), responsiveness (effect size), minimal important difference (MID), minimal important change (MIC), and floor and ceiling effects of the EQ-5D-5L were determined. To test discriminative ability, EQ-5D-5L utility indices of patients who were in a patient acceptable symptom state (PASS) or not at follow-up were compared using the Mann–Whitney *U* test.

**Results:**

We included 153 RCR, 150 TSA, and 151 CMC I patients. The EQ-5D-5L utility index correlated with the OSS (*r* = 0.73), SPADI (*r* = − 0.65) and brief MHQ (*r* = 0.61). The effect sizes were 1.3 (RCR and CMC I group) and 1.1 (TSA). The MID and MIC ranged from 0.027 to 0.209. Ceiling effects were found. The EQ-5D-5L utility index differed significantly between patients being in a PASS versus patients who were not in a PASS.

**Conclusion:**

The EQ-5D-5L utility index shows good construct validity, responsiveness and discriminative ability in patients after arthroscopic RCR, TSA and CMC I arthroplasty and is suitable to quantify quality of life.

**Clinical trial registration:** This auxiliary analysis is part of a primary study that was originally registered at ClinicalTrials.gov (NCT01954433) on October 1, 2013.

**Supplementary Information:**

The online version contains supplementary material available at 10.1007/s11136-021-02849-7.

## Plain English summary

The EQ-5D-5L questionnaire is a common tool used to measure health-related quality of life. It is one of the shortest and simplest instruments used to monitor health. However, its performance (measurement properties) in upper-extremity orthopaedic conditions has not been fully evaluated, yet. In our analysis, we studied the performance of the ED-5D-5L in patients who had surgery for various shoulder or hand conditions. Patients completed the EQ-5D-5L and other questionnaires documenting functional scores before surgery, 6 months and 1 year after surgery. We found that the EQ-5D-5L has good measurement characteristics and is suitable for assessing general quality of life in our patients with shoulder or hand disorders.

## Introduction

At the upper extremity, there are three prevalent musculoskeletal disorders that can markedly affect patient quality of life (QoL): rotator cuff tears as a result of direct trauma or age-related degenerative changes, glenohumeral osteoarthritis and thumb carpometacarpal (CMC I) osteoarthritis. Surgical treatment for these three specific conditions includes rotator cuff repair (RCR), total shoulder arthroplasty (TSA) and CMC I arthroplasty, respectively, all of which lead to good functional restoration of the joint and increased QoL for affected patients [1–3].

Since economic evaluations of interventions for musculoskeletal disorders are increasingly important nowadays, a sound outcome measure for quantifying QoL and quality-adjusted life years (QALYs) is required. Quality of life assessments for the aforementioned patient groups are also increasing, yet there is a lack of knowledge regarding the performance of QoL measures such as the EQ-5D and just how adequate this tool, in particular, is in determining health-related QoL.

For musculoskeletal disorders of the upper extremity, there are only a few studies that have investigated the measurement properties of the EQ-5D. For patients with proximal humeral fractures and carpal tunnel syndrome, the EQ-5D shows good reliability and construct validity [4–7]. Responsiveness was found to be high in patients after various elective shoulder surgery procedures [8, 9], but only moderate to good after interventions targeting the hand [6, 10, 11].

The aim of our study was to investigate the measurement properties of the EQ-5D-5L utility index in patients after arthroscopic RCR, TSA and CMC I arthroplasty. In particular, we focused on evaluating the properties of construct validity, responsiveness, minimal important difference (MID), minimal important change (MIC), floor and ceiling effects and discriminative ability.

## Methods

This analysis forms part of a previously published prospective cost-utility study [12, 13], which was conducted in a Swiss orthopaedic hospital and approved by the local ethics committee. We adhered to the Strengthening the Reporting of Observational Studies in Epidemiology (STROBE) statement [14].

### Patients

The study included three cohorts of: 1. patients with a rotator cuff tear who underwent arthroscopic RCR, 2. patients with glenohumeral osteoarthritis and/or rotator cuff tear who received a TSA, and 3. patients with CMC I osteoarthritis who underwent CMC I arthroplasty. All patients gave written informed consent for participation.

### Assessments

**Fig. 1:**
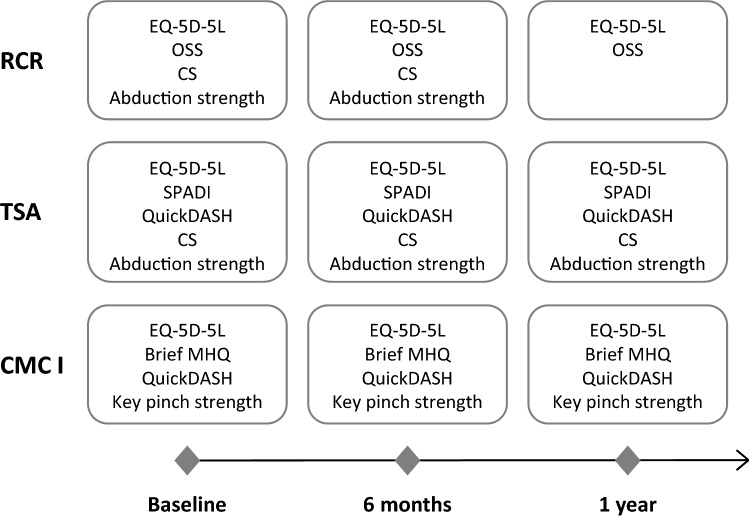
Outcome measures for the three patient groups at the different time points. *RCR* rotator cuff repair, *TSA* total shoulder arthroplasty, *CMC I* thumb carpometacarpal arthroplasty, *OSS* Oxford Shoulder Score, *CS* Constant Score, *SPADI* Shoulder Pain and Disability Index, *QuickDASH* short version of the Disabilities of Arm, Shoulder and Hand Questionnaire, *Brief MHQ* brief Michigan Hand Outcomes Questionnaire

Patients completed a set of German language questionnaires and underwent a clinical examination before surgery (i.e. baseline), and 6 months and 1 year post-surgery. RCR patients were not required to attend a clinical examination at 1 year. The timing of the following outcome measures is shown in Fig. 1.

After completion of the EQ-5D-5L [15], the 5 responses of the descriptive system were converted into an utility index using the EQ-5D-5L value set for Germany, which ranges from − 0.66 (lowest) to 1 (highest QoL) [16].

RCR patients completed the condition-specific Oxford Shoulder Score (OSS) [17], which ranges from 0 (worst) to 48 (best outcome) based on subjective levels of pain and impairment during activities of daily living.

Shoulder function of TSA patients was assessed using the Shoulder Pain and Disability Index (SPADI) [18], where scores range from 0 to 100 with higher scores indicating greater disability.

CMC I patients completed the brief Michigan Hand Outcomes Questionnaire (brief MHQ) for measuring hand function [19]. The score ranges from 0 to 100 with higher scores indicating better hand function.

TSA and CMC I patients also reported their function of the upper extremity by completing the short version of the Disabilities of the Arm, Shoulder and Hand questionnaire (QuickDASH) [20]. The QuickDASH yields a summary score between 0 and 100 with 0 indicating the best score or the lowest level of disability.

At both follow-ups, all patients were asked about their perceived change regarding their QoL, as has been proposed in the literature [21–23]: “In reference to your quality of life, do you feel *much better/slightly better/unchanged/slightly worse/much worse* compared to before the surgery?”

The two shoulder cohorts underwent clinical examinations for muscle strength in 90° abduction measured with a spring balance (Pesola AG, Schindellegi, Switzerland) and overall shoulder function based on the Constant Score (CS) [24]. The latter objectively measures range of motion, strength, pain and use of the shoulder in performing daily activities to yield a summary score between 0 (worst) and 100 (best outcome).

The clinical examination for CMC I patients included key pinch strength as measured in a standardized sitting position using a pinch gauge (B&L Engineering, Santa Ana, California, United States of America).

### Statistics

Standard descriptive statistics with means and standard deviations (SDs) were presented. To test for changes in outcomes between baseline and the 6-month and 1-year follow-ups, we used ANOVA for repeated measures. For assessing the clinical outcomes of the RCR group only recorded at 6 months, the Wilcoxon signed-rank test was used. As there were only a few missing data, they were not replaced.

The evaluation of measurement properties was based on the definitions and recommendations of the COnsensus-based Standards for the selection of health status Measurement INstruments (COSMIN) group [21, 25, 26].

*Construct validity* was tested with the following predefined hypotheses using the Pearson’s correlation coefficient (r):

- There is at least a moderate correlation of larger than 0.6 between the EQ-5D-5L utility index and OSS in the RCR group.
- There is at least a moderate negative correlation of larger than -0.6 between the EQ-5D-5L utility index and SPADI in the TSA group.
- There is at least a moderate correlation of larger than 0.6 between the EQ-5D-5L utility index and brief MHQ score in the CMC I group.

*Responsiveness* was evaluated by testing the predefined hypothesis that the effect size (Cohen’s d [27]) of the EQ-5D-5L utility index is greater than or equal to 0.8 in all three cohorts. This measurement property was calculated for the 1-year follow-up.

For *interpretability,* we calculated the minimal important difference (MID), which evaluates the smallest differences between patients or groups that are considered important [28]. The MID was determined by the difference in the EQ-5D utility index of patients who indicated a *slightly better* QoL after surgery and those with an *unchanged* QoL. Furthermore, the minimal important change (MIC), which defines the smallest change that patients consider important [28], was calculated using the following three methods: 1. a distribution-based method in which the MIC is equivalent to 0.5*SD [21, 29, 30], 2. an anchor-based approach using the mean change method and 3. another anchor-based approach using receiver operating characteristics (ROC). For the second method, the question about perceived change in QoL at follow-up compared with the baseline status was used as the anchor; the MIC was reflected by the mean change in the EQ-5D-5L utility index for those patients who considered themselves as feeling slightly better than before the surgery. For the ROC method, the 5-point Likert scale anchor question was transformed to a dichotomous scale with patients who had answered *much better* or *slightly better* being allocated to the improved group, and those with *unchanged*, *slightly worse* or much *worse* answers allocated to the comparison group of unimproved participants. The MIC was determined by finding the optimal cut-off point on the ROC curves, namely the point at which (1-sensitivity) + (1-specificity) was smallest [21].

*Floor and ceiling effects* at 1 year were noted if more than 15% of the patients achieved the lowest or highest possible scores [31].

To test the *discriminative ability* of the EQ-5D-5L, patients were divided into two groups based on whether they were either in a patient acceptable symptom state (PASS) at follow-up or not. The PASS is a patient’s subjective rating on whether they feel good and are satisfied with their current health status or not. Statistically, it is the value in a score beyond which patients consider themselves well [32]. For the RCR group, patients were categorized based on the OSS score. Christie et al. [33] published the PASS value of 26 for the OSS based on the former scoring system, which now corresponds to a value of 34 using the current OSS scoring method that we applied in this analysis [17]. The TSA patients were distributed based on the PASS value of 33.7 for SPADI [33], and CMC I patients based on the PASS value of 70 points for the brief MHQ [34]. The Mann–Whitney *U* test was used to test for differences in the EQ-5D-5L utility index for patients in either a PASS or not.

## Results

**Fig. 2:**
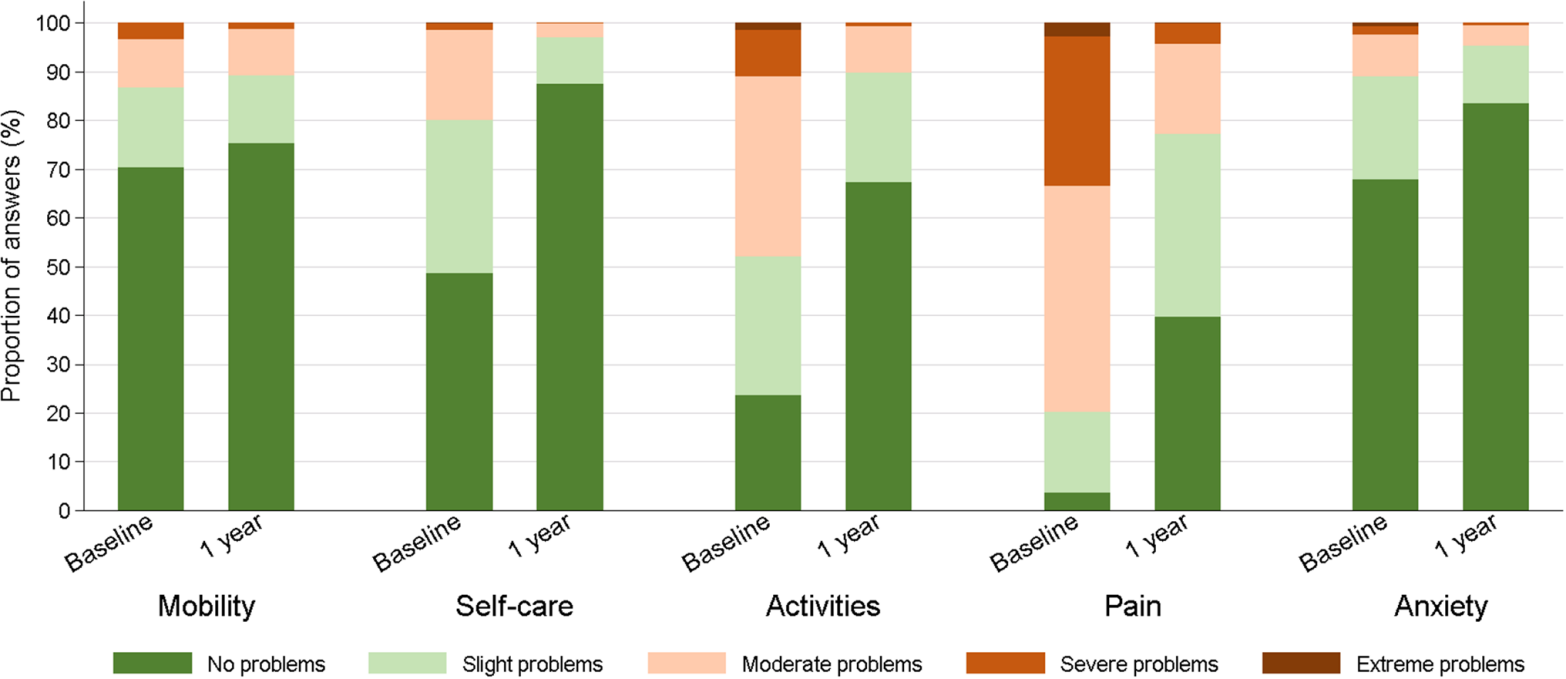
Dimension-specific results of the EQ-5D-5L for all patients at baseline and 1-year follow-up

From a total of 454 included patients, there were 153 in the RCR group, 150 in the TSA group and 151 in the CMC I group (Table 1). At the 1-year follow-up, the EQ-5D-5L utility indices of 148 RCR, 148 TSA and 150 CMC I patients were available. At baseline, the most affected EQ-5D-5L dimension for all patients was pain. Eighty percent of the patients indicated that they had moderate pain or worse (Fig. 2). For all patients, the mean baseline EQ-5D-5L utility index increased from 0.69 (SD 0.22) to 0.89 (SD 0.15) at 6 months and 0.90 (SD 0.13) at 1 year (*p* ≤ 0.001). There was also a significant increase in all outcome measures for each group, except key pinch strength (Table 2).

**Table 1:** Patient characteristics

	All	RCR	TSA	CMC I
Patients; *n*	454	153	150	151
Age at surgery; mean (SD)	64 (10)	57 (8)	71 (9)	65 (8)
Gender, female; *n* (%)	259 (57)	56 (36)	86 (57)	117 (77)
Type of surgery; * n* (%)*				
Arthroscopic rotator cuff repair		153 (100)		
Anatomical TSA			47 (31)	
Reverse TSA			103 (69)	
Simple trapeziectomy				10 (7)
Trapeziectomy with LRTI (autograft)				127 (84)
Trapeziectomy with LRTI (allograft)				7 (5)
CMC I pyrocardan implant				6 (4)
Trapeziectomy with absorbable gelatine sponge				1 (1)

*RCR* rotator cuff repair, *TSA* total shoulder arthroplasty, *CMC I* thumb carpometacarpal joint arthroplasty, *LRTI* ligament reconstruction and tendon interposition

*Percentages may differ from 100 due to rounding errors

**Table 2:** Outcome measures at baseline, 6 months and 1 year stratified by intervention group. Means and standard deviations are shown

	RCR	TSA	CMC I
EQ-5D-5L utility index [− 0.66 to 1]^a^			
Baseline	0.71 (0.23)	0.68 (0.23)	0.69 (0.19)
6 months	0.91 (0.14)	0.88 (0.15)	0.87 (0.15)
1 year	0.94 (0.11)	0.89 (0.15)	0.88 (0.11)
* p*-value	≤ 0.001	≤ 0.001	≤ 0.001
Oxford Shoulder Score [0 to 48]^a^			
Baseline	27 (8)		
6 months	42 (7)		
1 year	44 (5)		
* p*-value	≤ 0.001		
SPADI [0 to 100]^b^			
Baseline		62 (20)	
6 months		18 (18)	
1 year		15 (18)	
* p*-value		≤ 0.001	
Brief MHQ [0 to 100]^a^			
Baseline			49 (15)
6 months			77 (19)
1 year			83 (16)
* p*-value			≤ 0.001
QuickDASH [0 to 100]^b^			
Baseline		48 (17)	47 (16)
6 months		22 (18)	24 (19)
1 year		20 (17)	20 (17)
* p*-value		≤ 0.001	≤ 0.001
Constant Score [0 to 100]^a^			
Baseline	48 (17)	35 (15)	
6 months	69 (14)	68 (13)	
1 year		71 (15)	
* p*-value	≤ 0.001	≤ 0.001	
Abduction strength [kg]			
Baseline	4.0 (4.0)	1.0 (3.0)	
6 months	7.0 (3.0)	5.0 (3.0)	
1 year		5.6 (2.9)	
* p*-value	≤ 0.001	≤ 0.001	
Key pinch strength [kg]			
Baseline			3.9 (2.0)
6 months			3.4 (1.5)
1 year			3.9 (1.7)
* p*-value			0.15

*p*-values report the results of ANOVA for repeated measures (two follow-ups) or the Wilcoxon signed-rank test (one follow-up)

*RCR* rotator cuff repair, *TSA* total shoulder arthroplasty, *CMC I* thumb carpometacarpal joint arthroplasty, *SPADI* Shoulder Pain and Disability Index, *Brief MHQ* brief Michigan Hand Outcomes Questionnaire, *QuickDASH* short version of the Disabilities of the Arm, Shoulder and Hand questionnaire

^a^Higher values indicate better function

^b^Higher values indicate worse function

The EQ-5D-5L utility index showed moderate to high correlations with the OSS (*r* = 0.73), SPADI (*r* = − 0.65) and brief MHQ (*r* = 0.61) (Tables 3, 4 and 5).

**Table 3:** Pearson's correlation coefficients for the RCR group

	EQ-5D-5L utility index	OSS	CS
Oxford Shoulder Score (OSS)	0.73		
Constant Score (CS)	0.64	0.81	
Abduction strength	0.38	0.53	0.82

All correlations are significant with *p* < 0.001

**Table 4:** Pearson's correlation coefficients for the TSA group

	EQ-5D-5L utility index	QuickDASH	SPADI	CS
QuickDASH	− 0.67			
SPADI	− 0.65	0.86		
Constant Score (CS)	0.61	− 0.80	− 0.90	
Abduction strength	0.41	− 0.61	− 0.65	0.83

All correlations are significant with *p* < 0.001

*QuickDASH* short version of the Disabilities of the Arm, Shoulder and Hand questionnaire, *SPADI* Shoulder Pain and Disability Index

**Table 5:** Pearson's correlation coefficients for the CMC I group

	EQ-5D-5L utility index	QuickDASH	Brief MHQ
QuickDASH	− 0.64		
Brief MHQ	0.61	− 0.87	
Pinch grip	0.22	− 0.34	0.30

All correlations are significant with *p* < 0.001

*QuickDASH* short version of the Disabilities of the Arm, Shoulder and Hand questionnaire, *brief MHQ* brief Michigan Hand Outcomes Questionnaire

Responsiveness of the EQ-5D-5L was high in all three groups with effect sizes of 1.3 (RCR and CMC I group) and 1.1 (TSA) (Table 6). All condition-specific patient-reported outcomes showed higher effect sizes than the EQ-5D-5L.

**Table 6:** Responsiveness shown by effect sizes of the outcome measures stratified by intervention group

	RCR	TSA	CMC I
EQ-5D-5L utility index	1.3	1.1	1.3
Oxford Shoulder Score	2.4		
SPADI		2.5	
Brief MHQ			2.2
QuickDASH		1.7	1.6
Constant Score	1.4*	2.4	
Abduction strength	0.6*	1.6	
Key pinch strength			0.1

*RCR* rotator cuff repair, *TSA* total shoulder arthroplasty, *CMC I* thumb carpometacarpal joint arthroplasty, *SPADI* Shoulder Pain and Disability Index, *Brief MHQ* brief Michigan Hand Outcomes Questionnaire, *QuickDASH* short version of the Disabilities of the Arm, Shoulder and Hand questionnaire

*Calculated using the 6-months follow-up

The MID and MIC values, which ranged from 0.027 to 0.209, varied depending on the intervention group and also the calculation method for the latter (Table 7). With the ROC method, the area under the curve (AUC) values were 0.75, 0.69 and 0.59 for RCR, TSA and CMC I patients, respectively.

**Table 7:** Minimal important difference (MID) and minimal important change (MIC) of the EQ-5D-5L utility index calculated by different methods

	RCR	TSA	CMC I
MID	0.048	0.094	0.027
MIC—distribution	0.098	0.102	0.090
MIC—mean change	0.161	0.183	0.209
MIC—ROC	0.130	0.058	0.166

*RCR* rotator cuff repair, *TSA* total shoulder arthroplasty, *CMC I* thumb carpometacarpal joint arthroplasty, *ROC* receiver operating characteristic curve

One year after surgery, the highest possible EQ-5D-5L utility index of 1.0 was found in 54%, 36% and 17% of the RCR, TSA and CMC I patients, respectively, indicating a ceiling effect. The prevalence of the highest possible score in the disease-specific questionnaires was 34% of the OSS in the RCR group, 9% for the SPADI in the TSA group and 17% for the brief MHQ in the CMC I group. No floor effects for the EQ-5D-5L utility index (0%) were found in any group at any time point.

For all three populations, the EQ-5D-5L utility index differed significantly between the patients in a PASS versus those who were not, which indicates the good discriminative ability of this QoL outcome measure (*p* ≤ 0.001, Table 8).

**Table 8:** Discriminative ability of the EQ-5D-5L utility index for comparing patients in an acceptable symptom state (PASS) at any follow-up and those who are not

	RCR		TSA		CMC I	
	*n*	EQ-5D-5L utility index, mean (SD)	*n*	EQ-5D-5L utility index, mean (SD)	*n*	EQ-5D-5L utility index, mean (SD)
PASS	274	0.947 (0.078)	253	0.909 (0.133)	227	0.899 (0.116)
no PASS	24	0.663 (0.217)	43	0.741 (0.164)	73	0.800 (0.159)
*p*-value		≤ 0.001		≤ 0.001		≤ 0.001

*p*-values report the results of the Mann–Whitney *U* test for differences in the EQ-5D-5L utility index for patients in a PASS or not

*RCR* rotator cuff repair, *TSA* total shoulder arthroplasty, *CMC I* thumb carpometacarpal joint arthroplasty, *n* number of patients

## Discussion

The analysis of EQ-5D-5L utility index measurement properties revealed overall good construct validity, responsiveness and discriminative ability in patients after arthroscopic RCR, TSA and CMC I arthroplasty. However, the questionnaire shows considerable ceiling effects 1 year after surgery. The calculations of MID and MIC yielded inconsistent results with values varying from 0.027 to 0.209.

### Validity

All three hypotheses for testing construct validity were confirmed. Correlations between EQ-5D-5L and the disease-specific questionnaires, which measure joint-specific functional outcomes, were moderate to high in our three groups. Previous reports presented moderate (*r* = 0.57) and high correlations (*r* = 0.77) for the OSS versus EQ-5D three-level questionnaire [35] and EQ-5D-5L [36], respectively, in patients with shoulder disorders including rotator cuff tears, which also indicates good construct validity. Although it is unclear which EQ-5D version was examined, Dabija and Jain [37] found a negative correlation between the EQ-5D and SPADI in patients with rotator cuff tears; their correlation coefficient of -0.66 is similar to our value for TSA patients.

The correlation between the EQ-5D-5L and hand-specific brief MHQ was slightly lower in comparison to QoL versus the shoulder scores, yet closely matches the correlation established for patients with Dupuytren’s disease (*r* = 0.60) [10]. Further studies investigated correlations of the EQ-5D-5L and the original full MHQ and presented coefficients of 0.5 and 0.63 for patients with carpal tunnel syndrome [6] and various hand or wrist conditions [38], respectively. In general, the moderate correlations presented in this study and the aforementioned articles are due to the fact that the constructs measured by the EQ-5D-5L (i.e. quality of life) differ from the disease-specific patient-reported outcome measures (PROMs) that specifically focus on measuring shoulder or hand function.

### Responsiveness

Responsiveness of the EQ-5D-5L was high for all three groups with effect sizes of 1.1 and 1.3. Consequently, our predefined hypothesis that the effect sizes of the EQ-5D-5L utility index would be greater than or equal to 0.8 in all three cohorts was confirmed. A similar effect size of 1.186 was reported for EQ-5D (version unknown) assessments of patients after TSA [8]. Evans et al. [9] and Olerud et al. [4] also revealed high responsiveness of the EQ-5D-3L in patients after RCR, TSA and with a proximal humeral fracture. Although our study showed high responsiveness of the EQ-5D-5L after CMC I arthroplasty, this aspect was only low to moderate for other hand surgery interventions; the effect size was 0.25 in patients treated for Dupuytren’s disease [10] and 0.5 for patients after carpal tunnel release [6].

Responsiveness of the EQ-5D-5L in our three cohorts was expectedly lower than that noted for other disease-specific PROMs, as confirmed by the working groups of Evans, Hensler, Jain and Marti [6, 8–10]. This indicates that disease-specific PROMs are more suitable for assessing the treatment effect in these specific patient groups and should be used as outcome measures when subjective joint function is of primary interest. However, QoL significantly increased for all our patients 1 year after surgery and the QoL scores exceeded the EQ-5D-5L norm values, which have been determined for different age groups and are 0.87 for RCR patients and 0.85 for TSA and CMC I patients [39].

### Interpretability

The calculation of the MID and MIC yielded different values with large variances depending on the calculation method applied. Therefore, these inconsistent results should be considered with caution. A MIC of 0.09 has been calculated for patients after carpal tunnel release [6] and a simulation-based study revealed a MID of 0.083 for the German value set of the EQ-5D-5L independent of a specific disease or intervention [40]. Earlier published MIDs were 0.074 [41] or equivalent to half of a SD of the baseline score [30]. The latter study employed a distribution-based approach, which only considers the variance of the data and not the patient’s perspective. If possible, anchor-based approaches are preferred for MID and MIC calculations, which include a definition of what is considered as “minimally important” by the patient [42]. We used multiple methods to draw a comprehensive picture of the MIC estimate, as has also been suggested by Crosby et al. [43].

In the literature, ceiling effects of the EQ-5D-5L utility index ranging from 16 to 57% have been reported [4–6, 9]. Furthermore, we found a considerable ceiling for the EQ-5D-5L utility index at follow-up, at least for the RCR and TSA patients. However, ceiling also occurred in the disease-specific questionnaires for the RCR and CMC I patients, indicating that the patients’ health status 1 year after surgery is very good. Therefore, the high EQ-5D-5L utility indices can be partly attributed to the good health status of the patients and not to a ceiling effect in general. [4–6, 9].

### Limitations

As this analysis of measurement properties is a secondary analysis of a clinical trial, our patients did not complete the questionnaire twice within a short time frame, which precludes the calculation of test–retest reliability and measurement error. Furthermore, MID and MIC values differed considerably for all three groups depending on the calculation method because only a few patients indicated that their QoL was unchanged or worse than before surgery. This situation resulted in insufficient AUC values, particularly for the TSA and CMC I groups, and indicates an unreliable MIC; the number of unimproved patients must be higher to draw reliable conclusions about the MID and MIC. To determine the MIC, we used only one retrospective anchor question, which might have introduced recall bias, as patients might not exactly remember the preoperative status of the previous year [44].

## Conclusion

The EQ-5D-5L utility index shows good construct validity, responsiveness and discriminative ability in patients after arthroscopic RCR, TSA and CMC I arthroplasty. Despite the ceiling effects, the EQ-5D-5L seems to be a suitable tool for quantifying QoL in these patients, which is necessary to calculate quality-adjusted life years for cost-utility analyses.

## Supplementary Information

Below is the link to the electronic supplementary material.

Supplementary file1 (DOCX 29 kb)

## Data Availability

Data will be made available upon request.

## Code availability

The code will be made available upon request.
